# A new congenital cleft palate New Zealand rabbit model for surgical research

**DOI:** 10.1038/s41598-021-83400-z

**Published:** 2021-02-16

**Authors:** Haoyue Liu, Lingling Pu, Chialing Tsauo, Xiaoming Wang, Qian Zheng, Bing Shi, Chenghao Li

**Affiliations:** 1 State Key Laboratory of Oral Diseases, National Clinical Research Center for Oral Diseases, Department of Oral Maxillofacial Surgery, West China School of Stomatology, Sichuan University, Chengdu, 610041 People’s Republic of China; 2 Department of Cleft Lip and Palate Surgery, West China Stomatological Hospital, Sichuan University, No. 14, Section 3, Ren Min Nan Road, Chengdu, 610041 People’s Republic of China

**Keywords:** Anatomy, Diseases, Medical research, Risk factors

## Abstract

Cleft palate repair is a challenging procedure for cleft surgeons to teach, and in research, it can be difficult to evaluate different techniques and develop new treatments. In this study, a congenital cleft palate New Zealand rabbit model has been described and could be beneficial in future studies concerning cleft palate repair. Pregnant New Zealand rabbits received 1.0 mg dexamethasone injection intramuscularly once a day from the 13th gestation day (GD13) to GD16. On GD31. Newborn rabbits were delivered by cesarean sections, fed with a standardized gastric tube feeding method, and divided into two groups. The rate of survival and the incidence of cleft palate was calculated. Weight, appearance, behavior, maxillary occlusal view, and regional anatomic and histological comparisons were recorded within 1 month after birth. Infants from the two groups with similar physiological conditions were selected for continuous maxillofacial and mandibular Micro-CT scan and three-dimensional reconstruction analysis. Ten pregnant rabbits gave birth to 48 live infants. The survival and cleft palate rates were 65.6% and 60.4% respectively. Both groups survived over 1 month with no difference in weight, appearance, and behavior. The cleft type was stable, and anatomical defects, histological characteristics, and nasal-maxillary abnormalities of the cleft were similar to those of humans. There was no statistically significant difference in maxillary and mandible development between the two groups within one month after birth. This congenital cleft palate model is considered to have more research possibilities with efficient cleft induction, reliable feeding methods, stable anatomical defects, and maxillofacial development similar to those seen in humans.

## Introduction

Cleft palate repair is a challenging procedure for cleft surgeons to teach, and in research, it can be difficult to evaluate different techniques and develop new treatments, especially treatment outcome evaluations. The optimal design, time, and execution of this surgical operation are difficult to emulate in studies, thus, animal models are essential for addressing these issues.

The animal models applied in the study of cleft palate are divided into two types: (1) those induced by surgical operation, and (2) congenital cleft. In those that are prepared surgically, there is difficulty simulating the physiological structures of the congenital cleft, as well as the associated developmental characteristics of the maxillofacial region^1,2^. Additionally, the operation itself can also become another variable in the experimental results, therefore this is not a reliable treatment model. It is generally believed that large congenital animal models are ideal, such as dogs, sheep, and primates, as the results yielded may be more comparable to humans. However, the disadvantages of such models are that they may be time-consuming, costly, prone to miscarriage, and have unstable cleft types and unclear mechanisms. As well as that, there are few relevant studies involving large experimental models in cleft palate treatment^3–5^. Therefore, it may not always be appropriate to use large animals for the study of congenital cleft treatments. In small animal models, we have found that rabbits may have certain advantages over mice and rats. Rabbits are larger than rats, making the operation less technically difficult. They also have a docile disposition, greater surgical tolerance, a short gestation period (average 30 days), and larger litter size, allowing researchers to obtain a greater data pool to support the conclusion in a shorter experimental period^6^. As rabbits have been widely used as an experimental model in studies of maxillofacial development, local distraction osteogenesis, and palatal muscle regeneration in cleft lip and palate, there are sufficient relevant studies that may be used as a comparison. Furthermore, the pattern of bone accretion and muscle fiber composition in rabbits are similar to those of humans^7–10^. Among the currently published studies about postnatal cleft treatments, we found that all studies using rabbits as experimental models consisted of surgically induced clefts^11,12^. In this study, we evaluated the use of dexamethasone-induced New Zealand rabbits as experimental models for congenital cleft palate.

At the early stage of palatal shelf development, Walker injected several kinds of glucocorticoids into pregnant New Zealand rabbits for four consecutive days^13^. He found that the incidence of dexamethasone-induced cleft palate in the offspring was dose-dependent, but he did not build a new congenital cleft palate model for surgical research. In this study, we utilized New Zealand rabbits to develop a new congenital cleft palate model by using the phenotype Walker discovered. These New Zealand rabbits can be kept alive for more than 40 days with standardized gastric tube feeding methods, which ensures the growth of non-cleft palate and cleft palate rabbit infants under the same conditions. Based on the above, the establishment and characteristics of this model were standardized and summarized. We believe this is an appropriate model that could benefit surgical research of cleft palate treatments.

## Materials and methods

### Dexamethasone induction of cleft palate

**Figure 1:**
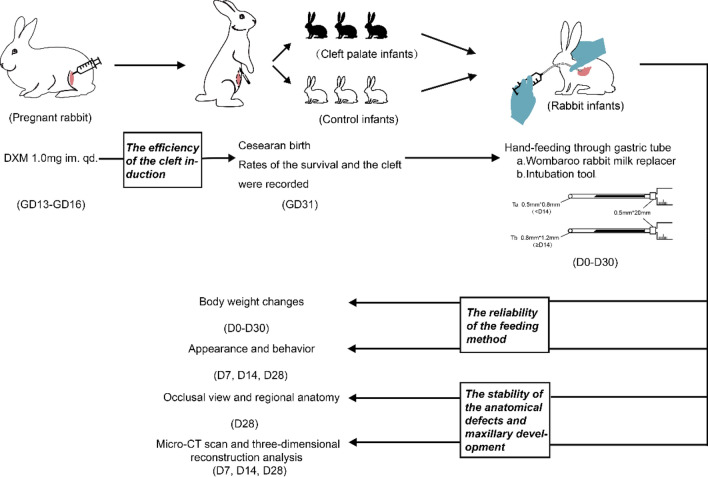
Procedures and descriptions of the congenital cleft palate rabbit model. *DXM* dexamethasone, *D* day (age of infants).

All experimental procedures on animals were in accordance with the National Institute of Health Guidelines for the Care and Use for Laboratory Animals and were approved by the Ethics Committee of West China College of Stomatology, Sichuan University (Protocol number: WCHSIRB-D-2017-090). Female New Zealand rabbits (Animal Center of Sichuan University, Chengdu, China) weighing 4.5–5.0 kg, and at 40 weeks of age were selected because of good fertility. Each female rabbit was kept in a separate cage, and the feeding room was kept quiet, clean, and ventilated with the relative humidity between 40 and 70%, and the temperature between 20 and 25 °C. Ten female New Zealand rabbits were cooped with males of the same strain at 5:00 p.m. and separated the next morning. Ten hours after mating was determined to be the fertilization time. On the 7th day after mating, we confirmed that all females were successfully pregnant. Dexamethasone (Dexamethasone sodium phosphate injection, 1.0 ml: 5.0 mg, TJYP Co., Ltd., Tianjin, China) was injected intramuscularly into the quadriceps of pregnant rabbits at 8:00 a.m. for 1.0 mg each day from GD13 to GD16. All the above operations should be performed gently and rapidly to reduce the disturbance to pregnant rabbits. To obtain newborn rabbits, standardized cesarean sections were performed on 10 pregnant rabbits under carbon dioxide gas anesthesia on GD31^14^. We cut open the uterus carefully, quickly removing all living infants and dead fetuses. Once the breathing of living infants was observed, the whole body was dried, and immediately transferred to the incubators at 30 °C. The number of those that survived and the occurrence of cleft palate was recorded, and the corresponding ratio was then calculated (Fig. 1).

### How to care for newborn rabbits

We referred to previous artificial feeding techniques for rabbits^15^, and at 4 h after birth, the feeding was started with the special rabbit milk powder (Wombaroo Food Products Co., Glen Osmond, Australia). The initial dose was 2 ml. The infants were then fed three times a day at 8:00, 14:00, and 20:00, in doses that were equal to 20% of their body weight. The preparation method of the milk powder for the rabbits is as follows: the ratio of water to powder at 0–3 days old is 5:1, 4:1 at 4–5 days old, and 3:1 at 6–7 days old; the milk should be heated and the temperature maintained at 37 °C. If the infant’s weight increases steadily after 1 week, the ratio of water to powder should be changed to 2:1. Plastic capillary tubes with an inner and outer diameter of 0.5 m × 0.8 mm (before 2 weeks old) and 0.8 mm × 1.2 mm (after 2 weeks old) were connected to a needle with a no.5 syringe (0.5 mm × 20 mm) for intubation (Fig. 1). The standardized gastric tube feeding method is as follows: wrap the baby rabbit's limbs in a clean towel with one hand, place your thumb and forefinger on the infant’s head and tilt it slightly toward its abdomen, then use the thumb and forefinger of the other hand to gently insert the tube from the corner of the infant's mouth, and gently push the tube into the infant's esophagus with the infant’s swallowing motion. Slight resistance may be felt during the process. If the tube enters the esophagus correctly, infants should have no obvious struggle and continue to swallow normally. When feeding, slowly push the milk and continue until the infant’s abdomen is slightly bulging. At 1 week old, the genital area of infants must be stimulated to make them pee at 8:00 p.m. every day. At 4 weeks old, both groups of young rabbits could start consuming soft foods. The humidity in the incubator should be 55–70%, and the temperature should be 28–30 °C before 2 weeks old and 25–28 °C after 2 weeks old.

### Evaluation of the model and statistical analysis

Bodyweight and behavioral observation, surgical anatomy, histological analysis (hematoxylin and eosin technique, H&E), and microfocus computed tomography (micro‐CT) were employed to evaluate the cleft model and present the soft tissue and craniofacial characteristics of individuals with cleft palate. All statistical analyses were performed using SPSS Statistics 23.0 software (IBM SPSS Inc, the USA). Indexes were presented as mean value, and the same index comparisons between two groups were tested using the Homogeneity of variance test and the one-way analysis of variance (ANOVA). A value of *p* < 0.05 was considered statistically significant.

## Results

### Bodyweight and behavior evaluation

**Figure 2:**
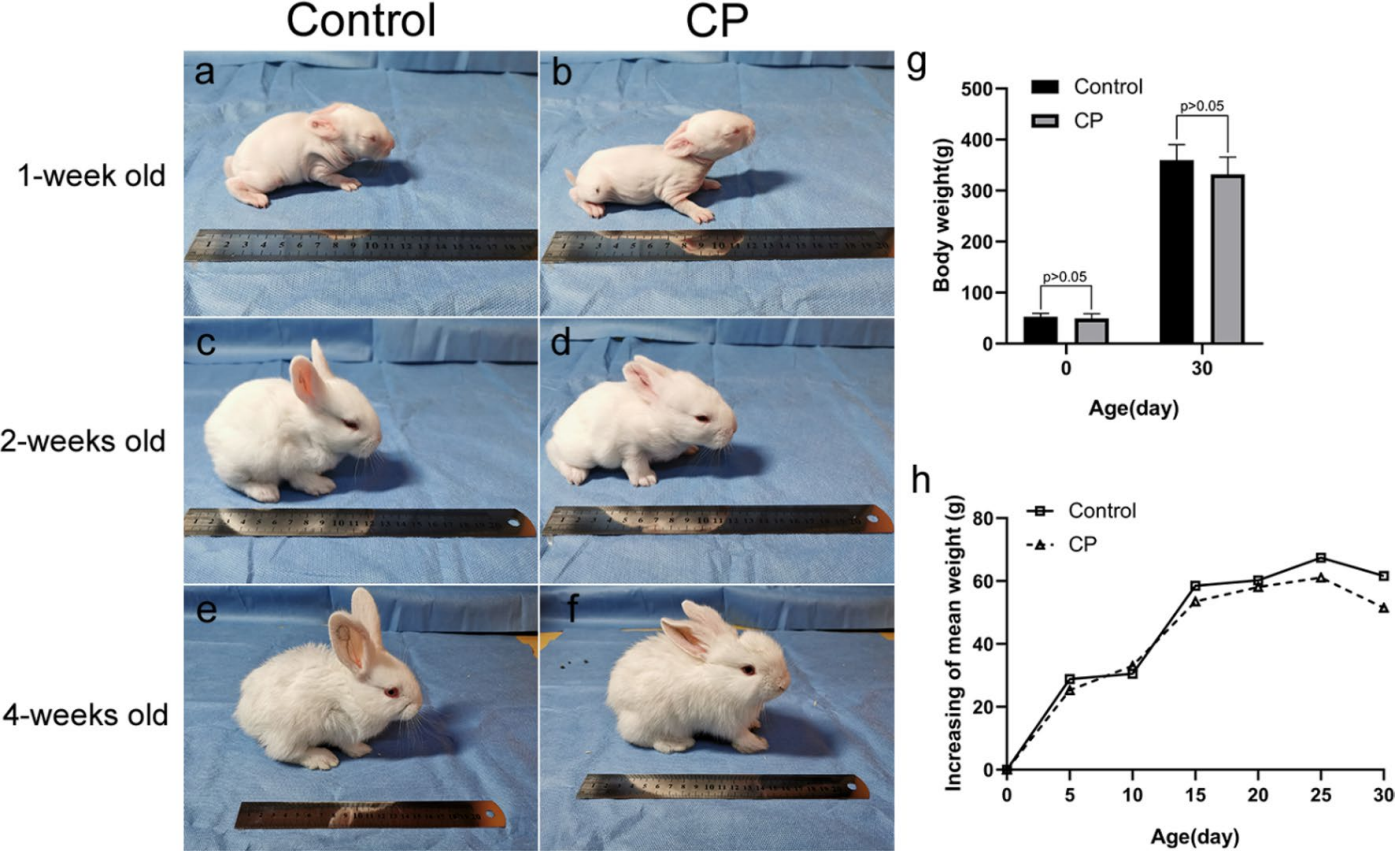
Appearance changes (left) of the control group (**a**, **c**, **e**) and the CP (cleft palate) group (**b**, **d**, **f**) at week 1 (**a**, **b**), 2 (**c**, **d**), and 4 (**e**, **f**), and the length of the ruler is 20 cm. The body weight changes and comparison (right) between the two groups (**g**, **h**). There was no significant difference in the weight of the two groups at birth and 1 month old (*p* > 0.05) (**g**). The change pattern of the mean weight gaining every 5 days was basically the same between the two groups within 1 month old (**h**).

**Figure 3:**
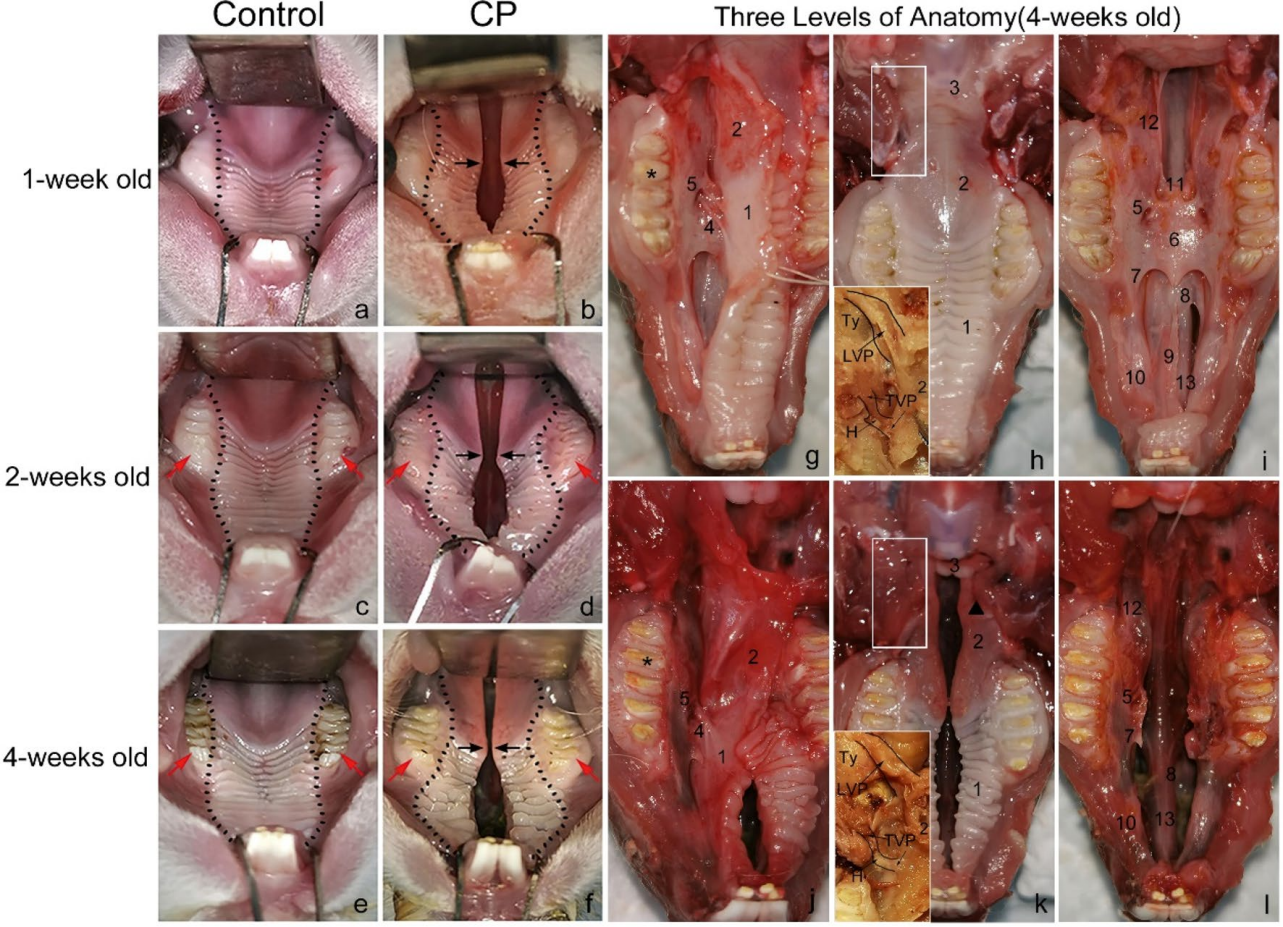
Occlusal views (left) of the control group (**a**, **c**, **e**) and the cleft palate group (**b**, **d**, **f**) at week 1 (**a**, **b**), 2 (**c**, **d**), and 4 (**e**, **f**). The dotted line represents the U-shaped palate contour in the control group and the hourglass-shaped palate in the cleft palate group. The black arrow indicates that the gap gradually narrowed at the junction of the hard and soft palate, but widened at the front of the hard palate. Notice that premolars and molars in the cleft showed slow eruption and dental dysplasia compared with those in the control (red arrow). Regional anatomic comparisons (right) of the control group (**g**, **h**, **i**) and the cleft palate group (**j**, **k**, **l**) at week 4. Three levels of anatomy: Parapharyngeal and soft palate mucosa and muscles after removing the mandible (**h**, **k**); Lateral sub-mucoperiosteal tissue surrounding the alveolar (**g**, **j**); Bony structure along the midline of the maxilla (**i**, **l**). Asterisk: the first molar. Black triangle: strip-shaped muscle at the margin of the crack. *C* control, *CP* cleft palate, *TVP* tensor veli palatini muscle, originating from the lateral surface of the pterygoid plate and turning around a curved process, *LVP* levator veli palatini muscle, arising from the lateral and posterior surface of tympanic bulla. *H* pterygoid hamulus, *Ty* tympanic bulla. 1—Hard palate, 2—soft palate, 3—epiglottic cartilage, 4—anterior palatine nerve, and blood vessel, 5—foramina palatina majora, 6—palatine bone plate, 7—palatine process of maxilla, 8—vomeronasal organ, 9—incisor bone, 10—premaxillary, 11—ala of the vomer, 12—pterygoid plate of the sphenoid bone, 13—nasal septum.

Ten pregnant rabbits gave birth to 48 live infants, with a survival rate of 65.6%. Among the surviving young rabbits, there were 29 cleft palate infants, with an incidence rate of cleft palate (60.4%) (Table 1). Embryos with cleft palate are more likely to be absorbed or die at birth than those without cleft palate. The litter size of each pregnant rabbit was different, but all were consistent with the normal production capacity of pregnant rabbits. After birth, every infant rabbit was identified as having a cleft palate or a normal palate and randomly assigned into one of two groups respectively, the cleft palate group (CP) and the control group (C), and each group contained 10 infant rabbits. Group-housed rabbits were identified with nontoxic paint marked on the head. The body weight of each infant rabbit was recorded every 5 days, and the physiological behavior was also observed every day (Table 2; Fig. 2). The frontal view and the maxillary occlusal view (under general anesthesia with a subcutaneous injection consisting of 0.6% pentobarbital, 6 ml/kg) of the rabbits were photographed at 1, 2, and 4 weeks old (Figs. 2, 3)**.** At over 1 month, there were no mortalities of the twenty infants included in both the control group and the cleft palate group. After birth, the weight of the rabbits in the two groups increased steadily, reaching the weight range of the SPF rabbits that were artificially reared in Syukuda’s study (Table 2)^15^. There was no significant difference in birth weight and 1-month-old weight between the two groups (*p* > 0.05). When comparing the patterns of weight increase every 5 days, the trend in that of the cleft group and the control group were the same. In both groups, hair began to appear on day 3, eyes opened on day 12, foraging was observed more frequently on day 14, and soft particles could be fed gradually starting from day 25. It was almost impossible to distinguish the infant rabbits in the cleft palate group from the control group solely by observing the appearance and behavior (Fig. 2).

**Table 1:** Frequency of survival and cleft palate in offspring of pregnant does given dexamethasone for 4 consecutive days during pregnancy.

Does*	No. of infants			No. of alive infants	
	Total	Alive	Dead/resorbed*	CP	Control
1	9	6	3	4	2
2	2	0	2	0	0
3	11	8	3	8	0
4	4	4	0	2	2
5	7	3	4	2	1
6	6	6	0	4	2
7	7	4	3	1	3
8	8	8	0	5	3
9	8	2	6	0	2
10	11	7	4	3	4
Total	73	48	25	29	19
Rate (%)	100.0	65.6	34.3	60.4	39.6

*The 10 pregnant rabbits were numbered from 1 to 10. *These include aborted infants and absorbed embryos.

**Table 2:** Bodyweight records of the two groups of rabbits every 5 days.

Infants	Alive/dead*	Bodyweight (g) at the age in days						
		0	5	10	15	20	25	30
C	10/0							
	Mean	52.8	81.6	112.1	170.6	230.8	298.2	359.8
	(SD)	(6.6)	(9.6)	(12.7)	(18.0)	(25.5)	(21.4)	(30.4)
	Max	63.8	94.0	130.8	191.0	270.0	320.8	408.7
	Min	43.5	60.7	87.7	135.2	189.8	259.1	311.9
CP	10/0							
	Mean	49.5	74.8	107.8	161.3	219.4	280.5	332.0
	(SD)	(9.1)	(11.7)	(11.1)	(19.7)	(24.6)	(31.4)	(33.8)
	Max	64.8	89.3	125.2	189.5	263.2	324.0	392.2
	Min	38.9	55.3	85.8	122.1	181.2	232.0	291.5
	*p*	0.364	0.294	0.508	0.362	0.436	0.208	0.069

Mean values, standard deviation (SD), the Max values, the Min values, and *p* values at each time point are shown for each group (C: control group; CP: cleft palate group).

*Number of deaths within 1 month of age.

### Anatomical evaluation of the model

Three rabbits, aged 4 weeks, were randomly selected from each of the two groups, sacrificed by intravenous air embolism, and fixed on the anatomical table. A midline neck incision was made to separate the connective tissue until the skin was stripped from the neck and above. After removing the submandibular gland on both sides, the posterior belly of the digastric muscle was dissected to its origin to expose the tympanic bulla, and to observe the origin and insertion of the levator veli palatini. By cutting off the mandibular ramus on both sides, the maxillary occlusal surface was exposed and the soft palate was dissected to observe the origin and distribution of the tensor veli palatini. By removing the hard and soft palate mucosa and parapharyngeal soft tissues, bone structures from the incisor to the pharynx were completely exposed so that defects in the midline of the maxilla could be observed (Fig. 3). From weeks 1–4, the contour of the palate in the control group was a steady “U” shape (Fig. 3a, c, e), while the palate of the rabbits in the cleft palate group gradually became an “hourglass” shape (Fig. 3b, d, f). In the cleft group, there was only one rabbit with an incomplete cleft palate, all the others had a complete cleft palate. The buccal inclination of the dental arch and slow eruption of teeth were observed (Fig. 3c–f). Regional anatomic comparisons showed lateral sub-mucoperiosteal tissue surrounding the alveolar (Fig. 3g, j). The soft palate of the cleft rested on the dorsal side of the epiglottic cartilage, and the palate muscles of both groups originated from the same anatomic site and were distributed in a similar way (Fig. 3h, k). In the cleft, the levator veli palatini muscles inserted anteriorly and ran parallel to the margins of the cleft, and the tensor veli palatini muscles diffused into the palatine aponeurosis medially and anteriorly. The location of the foramina palatina majora was in the soft palate mucosa and the palatal side of the first molar. From the incisors to the pharynx, the cleft palate group lost almost all its important bone base in the mid-maxillary line. The incisor bone and the hard palate bone plate in the cleft were missing, and the vomer and the sphenoid pterygoid bone plates were severely underdeveloped. The palatal process of the maxilla is presented as an irregular thin bone plate (Fig. 3i, l).

### Histological analysis of the model

**Figure 4:**
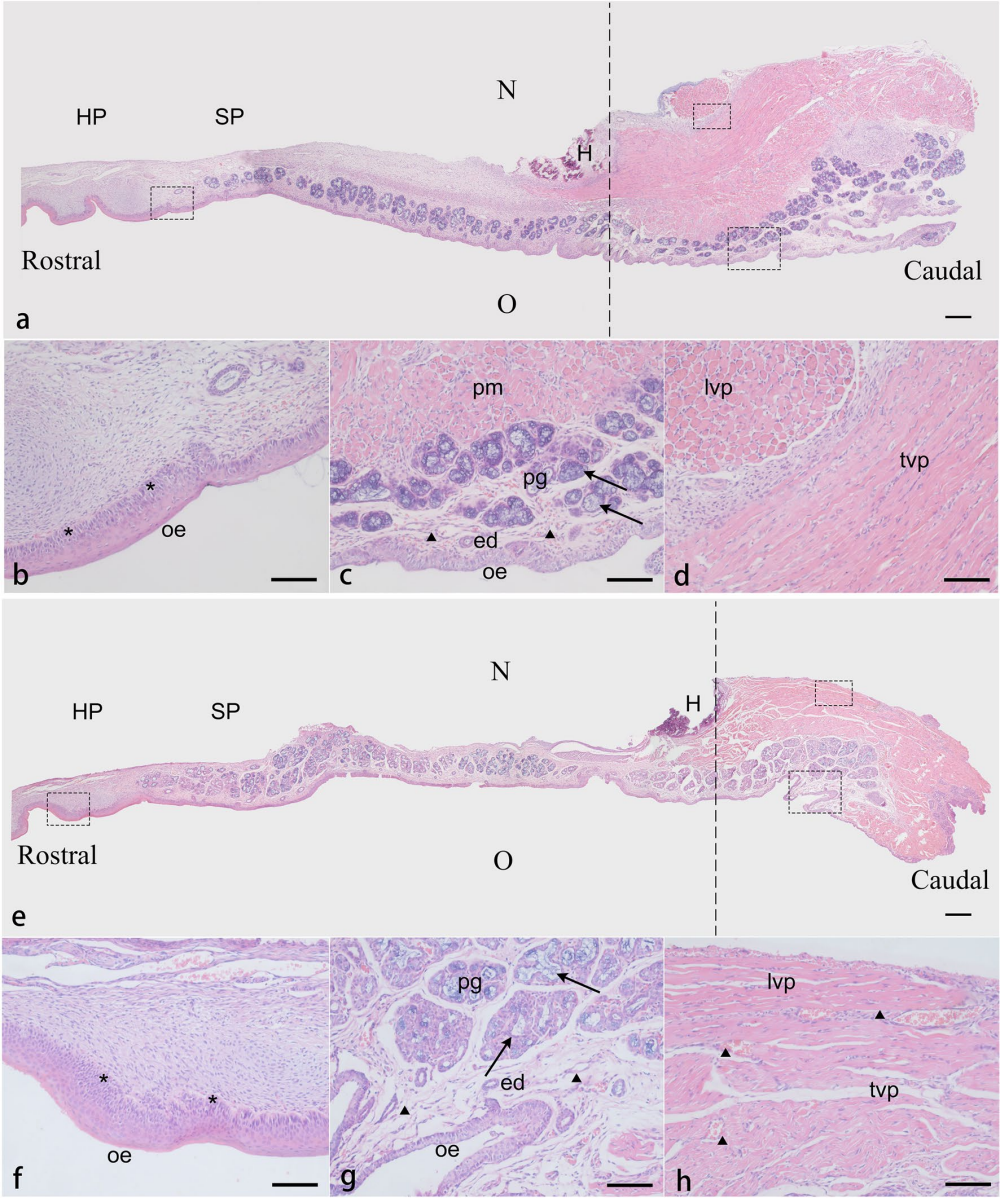
Histology view of the midsagittal sections of the palate in the control (**a–d**) and the cleft (**e–h**). Paraffin sections were cut at the level of the pterygoid hamulus (H) and stained with H&E. The dotted lines indicate the level of the coronal sections. (**a**, **e**) The cleft shows a reduction in the amount of nasal submucosal connective tissue in the hard palate and the anterior soft palate. Muscle tissues account for about the posterior second of the soft palate in the control, while about the third of the soft palate in the cleft. (**b**, **f**) The asterisk represents in the cleft the disordered arrangement and hyperplasia of the stratum basale cells in adjacent submucosa. (**c**, **g**) Arrows in the control show that the New Zealand rabbit palatal glands are mixed glands with serous cells surrounding mucous acini. In the cleft group, the degeneration and abnormal morphology of glandular tissue can be seen, and mucous acini are more common. Between a thin stratified squamous epithelium and palatal glands is an underlying loose connective which is thicker and looser in the cleft (the triangle). (**d**, **h**) The triangle in the cleft also shows the disordered muscle bundle arrangement and hyperplasia of connective tissue and blood vessels between muscle tissue. *HP* hard palate, *SP* soft palate, *N* nasal cavity, *O* oral cavity, *H* pterygoid hamulus, *oe* oral epithelium, *pm* palatal muscle, *pg* palatal gland, *ed* excretory duct, *lvp* levator veli palatini muscle, *tvp* tensor veli palatini muscle. Scale bars: **a**, **e** = 200 µm; **b**–**d**, **f–h** = 50 µm.

**Figure 5:**
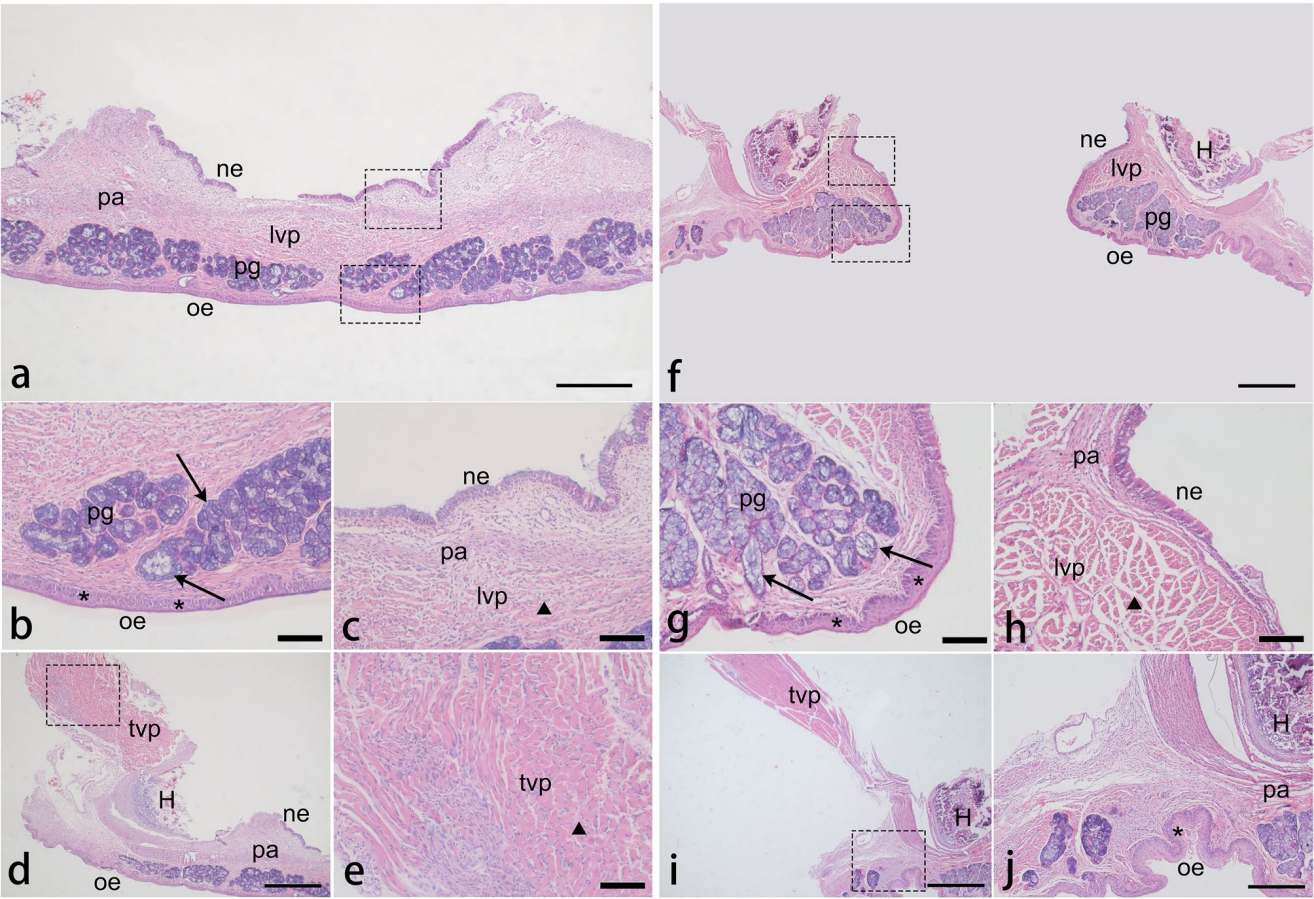
Histology view of the coronal sections of the palate in the control (**a**–**e**) and the cleft (**f–j**). Paraffin sections were cut at the level of the pterygoid hamulus (H) and stained with H&E. The soft palate stratigraphy can be seen, from the mesial side (**a**, **f**) to the distal side (**d**, **i**), from the nasal side (top) to the oral side (bottom). (**b**, **g**, **j**) The asterisk represents a thin stratified squamous epithelium in the control and a thickened stratum corneum in the oral epithelium of the cleft. What the arrow shows reconfirm the differences of the type and morphology of the palatal gland between the two groups. (**c**, **h**, **e**) The triangle shows the morphology of palatal muscle tissues in two groups and reconfirms the looser muscle bundle arrangement in the cleft. The levator veli palatini fibers in the control crossed the midline forming a muscle sling, while muscle bundles in the cleft present a cross-section morphology indicating that the levator veli palatini ran parallel to the edge of the cleft. The palatine aponeurosis can be seen, lying under the nasal epithelium, a pseudostratified ciliated columnar epithelium. (**d**, **i**, **j**) The tensor veli palatini muscle in both groups diffused into the palatine aponeurosis medially and anteriorly at the pterygoid hamulus. *ne* nasal epithelium, *oe* oral epithelium, *pa* palatine aponeurosis, *pg* palatal gland, *lvp* levator veli palatini muscle, *tvp* tensor veli palatini muscle, H: pterygoid hamulus. Scale bars: **a**, **d**, **f**, **i** = 200 µm; **j** = 100 µm; **b**, **c**, **e**, **g**, **h** = 50 µm.

Eight newborn rabbits (4 noncleft and 4 cleft) were randomly selected to assess cellularity and tissue organization of the palate. After euthanasia, the palates of the eight newborn rabbits were dissected, rinsed in 0.1 M phosphate-buffered saline (PBS, pH 7.4), and then fixed in 4% paraformaldehyde (PFA) for 24 h at 4 °C. After fixation, eight specimens of the palate were divided into two groups, group 1 (2 noncleft and 2 1cleft) for midsagittal sections and group 2 (2 noncleft and 2 cleft) for coronal sections, then dehydrated in a graded series of ethanol, and paraffin-embedded. Each specimen was further subdivided into six different comparable levels in average length and cut for 5-µm serial sections which would be routinely stained with H&E from each level^16^. The sections from two directions further confirmed the observations and supplemented what might have been missing. And we found that, at the pterygoid hamulus level, the histological characteristics of the palatal cells and tissues could be presented more comprehensively (Figs. 4, 5). The oral surface was covered by a keratinized stratified squamous epithelium which gradually thinned from the rostral side to the caudal side (Fig. 4a). In the cleft group, the stratum basale cells in the hard palate epithelium showed obvious hyperplasia and disordered arrangement, and there was a thicker stratum corneum in the soft palate epithelium compared with that of the control (Figs. 4b, f, 5b, g). Glandular tissues filled the axial part of the soft palate and were distributed in the oral side with distinct differences between the control and the cleft in the acinar type and morphology (Figs. 4c, g, 5b, g). The majority in the control were mixed glands with serous cells surrounding mucous acini, whereas separate and abnormal mucous acini were more common in the cleft. In addition to the degeneration of the glands, the submucosal connective tissue, including the excretory duct and blood vessels, showed a tendency of irregular hyperplasia between the glands and muscles in the cleft group (Fig. 4g, h). One of the main areas of the palate is the muscle tissue which accounts for about the posterior second of the soft palate in the control, while about the third of the soft palate in the cleft. In addition to changes in muscle mass, the local disordered and loose arrangement of muscle bundles were also observed (Fig. 4a, d, e, h). The spatial relationship of the levator veli palatini muscle, tensor veli palatini muscle, and palatine aponeurosis in both two groups was consistent with the anatomical observations. (Fig. 5a, c, d, f, h, i).

### Microfocus computed tomography (Micro‐CT) for imaging survey of the model

**Figure 6:**
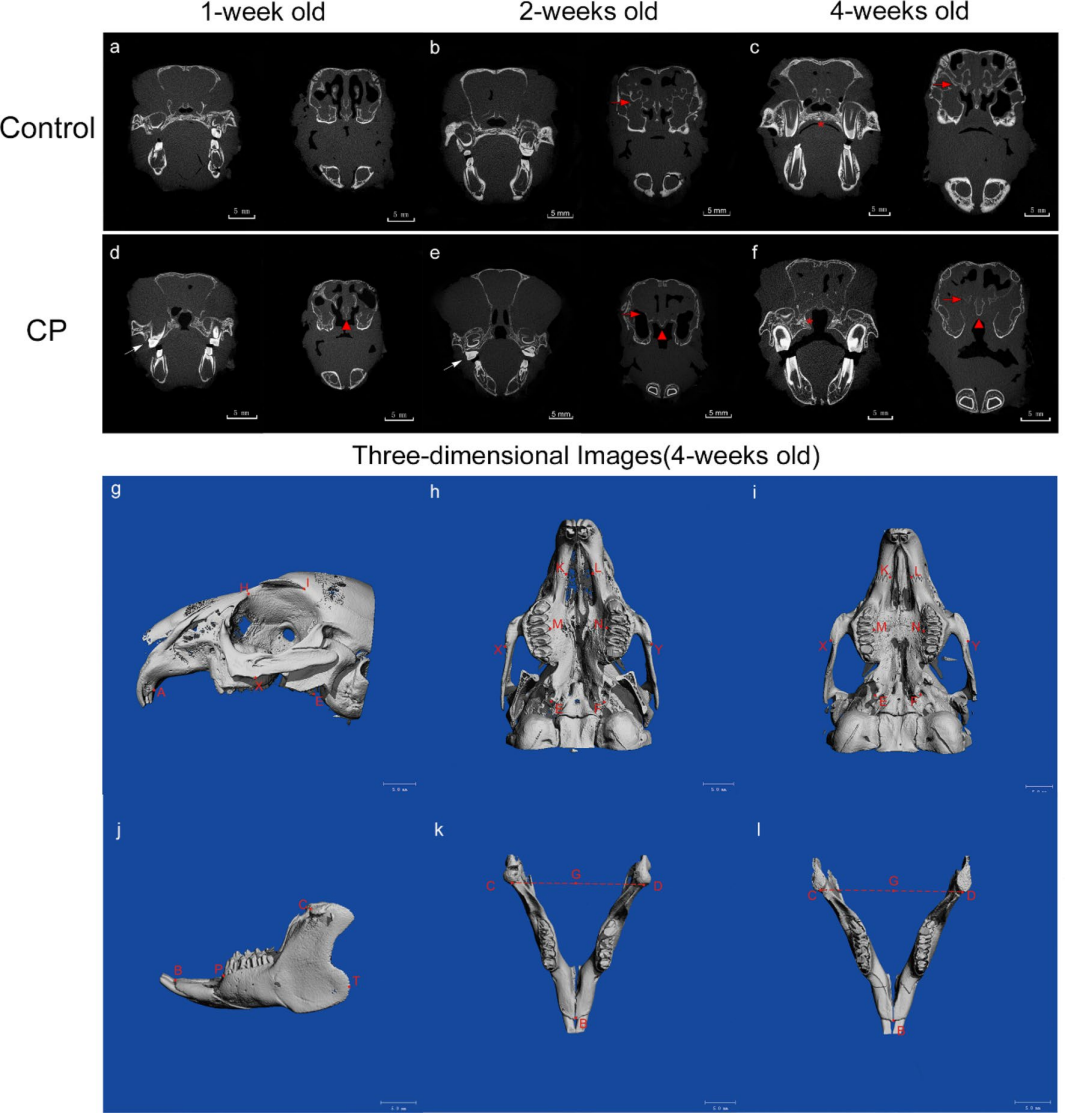
Oral and Maxillary views in Micro-CT sections (above) of the control group (**a–c**) and the cleft palate group (**d–f**) at week 1 (**a**, **d**), 2 (**b**, **e**), and 4 (**c**, **f**). The layers shown in the CT coronal section are at the anterior margin of the hard palate and the second premolar. The red arrow indicated that the development of turbinates and sinuses was insufficient in the cleft. The white arrow shows the abnormal position of the dental arch and the disordered occlusion. Notice the small vomer and septum of the cleft (the red triangle). The red asterisk indicates the different sites of bone development of the palatal process in the two groups. Occlusal and lateral views in three-dimensional reconstruction images with marking points (below) of the maxilla (**g**, **h**, **i**) and mandible (**j**, **k**, **l**) from the control group (**i**, **l**) and the cleft palate group (**h**, **k**) at week 4. Maxillary height: H1 (H–X), H2 (E–I); Maxillary length: L1 (A–X), L2 (A–E); Maxillary width: W1 (K–L), W2 (M–N), W3 (E–F), W4 (X–Y); Mandibular length: L3 (B–P), L4 (B–T); Mandibular height: L5 (B–C), L6 (B–G).

Three infants, weighing 45–50 g were randomly selected in each of the two groups, and their maxillofacial region was scanned using micro-CT (SCANCO VivaCT80, Switzerland) at 1, 2, and 4 weeks old under general anesthesia with subcutaneous injection (0.6% pentobarbital, 6 ml/kg). The voltage and current parameters were set to 70 kV and 114 μA (Fig. 6a–f). Three-dimensional reconstructions of the maxilla and mandible, markings of relevant points, and measurements of distance indexes were all performed on the Viva CT image workstation by the same person. The measured values of each distance index were used to compare the maxillary and mandibular development of the two groups at the same time point. The following marking points were used: A (the posterior alveolar process point of the maxillary incisors); H (the nasal process point); I (the point at the posterior supraorbital process of the frontal bone); K, L (the mesial margin point of the premaxillary-maxillary suture); X, Y (The lowest point of the masseter spine of the zygomatic process); M, N (the alveolar process point of the third premolar); E, F (the pterygoid hamulus point); B (the posterior alveolar process point of the mandibular incisors); P (the mesial alveolar process point of the first premolar); T (the extreme posterior endpoint of the mandible); C, D (the anterior edge point of the condyle); G (the midpoint of the line between point C and point D ) (Fig. 6g–l). These points were the reference for the maxillary and mandibular development of the New Zealand rabbits^17,18^. During the growth period in the cleft, the thin maxillary palatal process, short vomer, and the nasal septum in the cleft did not develop. Likewise, the dysplasia of the sinus structure and low bone density of surrounding bones did not improve (Fig. 6d–f). Only the distance between the bilateral pterygoid hamulus points at 1 week old was statistically significant between the two groups, and there was no statistically significant difference in the other maxillary indexes (Tables 3, 4). Most of the mean values of mandibular length index were lower in the cleft group compared with the control at all time points, but only the distance was a statistically significant difference between the incisor alveolar process point and the extreme posterior endpoint at 1 week old (Table 5).

**Table 3:** Statistical values for height and length measurements of the maxilla using computed tomography three-dimensional reconstructions.

	H1		*p*	H2		*p*	L1		*p*	L2		*p*
	C	CP		C	CP		C	CP		C	CP	
	Mean (SD)	Mean (SD)		Mean (SD)	Mean (SD)		Mean (SD)	Mean (SD)		Mean (SD)	Mean (SD)	
1-week	11.0567 (1.1852)	10.8833 (0.8343)	0.846	11.8433 (1.0624)	13.3067 (0.6257)	0.109	11.9300 (0.5820)	11.8900 (0.3242)	0.922	18.9867 (0.7575)	19.1533 (0.2765)	0.738
2-week	13.3500 (1.2401)	12.5767 (0.8639)	0.426	15.1800 (0.8802)	13.7467 (0.5164)	0.072	14.2067 (1.2816)	14.3400 (1.0776)	0.897	21.6767 (1.7256)	21.0700 (0.3913)	0.585
4-week	15.8433 (1.2036)	15.5967 (1.0226)	0.800	18.1000 (0.9283)	17.0600 (1.3273)	0.328	18.8200 (1.3928)	17.1033 (0.9286)	0.150	27.5667 (1.5685)	25.9400 (1.0817)	0.213

Mean values, standard deviation (SD), and *p* values of each distance index are shown for each group (C: control group; CP: cleft palate group) at each time point in development. Maxillary height: H1 (H–X), H2 (E–I); Maxillary length: L1 (A–X), L2 (A–E).

**Table 4:** Statistical values for width measurements of the maxilla using computed tomography three-dimensional reconstructions.

	W1		*p*	W2		*p*	W3		*p*	W4		*p*
	C	CP		C	CP		C	CP		C	CP	
	Mean (SD)	Mean (SD)		Mean (SD)	Mean (SD)		Mean (SD)	Mean (SD)		Mean (SD)	Mean (SD)	
1-week	3.0800 (0.2563)	3.3133 (0.1986)	0.281	7.2113 (0.7147)	6.6333 (0.3707)	0.280	5.4667 (0.2011)	6.3300 (0.2011)	0.016*	19.7433 (0.6341)	19.7000 (0.4709)	0.929
2-week	3.2767 (0.2303)	3.6633 (0.2665)	0.130	7.5900 (0.5009)	7.5367 (0.7514)	0.923	6.4233 (0.8800)	6.4500 (0.9283)	0.973	21.5800 (0.6630)	20.3267 (0.8452)	0.113
4-week	3.6233 (0.1779)	3.8500 (0.4513)	0.464	8.7567 (0.6982)	8.4967 (0.5486)	0.639	8.6833 (0.3067)	7.9200 (1.0530)	0.294	25.7433 (0.5330)	24.6333 (1.5769)	0.312

Mean values, standard deviation (SD) and *p* values of each distance index are shown for each group (*C* control group; *CP* cleft palate group) at each time point in development.

*p < 0.05. Maxillary width: W1 (K–L), W2 (M–N), W3 (E–F), W4 (X–Y).

**Table 5:** Statistical values for length and height measurements of the mandible using computed tomography three-dimensional reconstructions.

	L3		*p*	L4		*p*	L5		*P*	L6		*p*
	C	CP		C	CP		C	CP		C	CP	
	Mean (SD)	Mean (SD)		Mean (SD)	Mean (SD)		Mean (SD)	Mean (SD)		Mean (SD)	Mean (SD)	
1-week	6.4278 (0.4600)	5.8828 (0.2710)	0.152	21.2355 (0.7718)	19.4442 (0.6022)	0.034*	20.6654 (0.8981)	18.6727 (1.3528)	0.101	19.9595 (0.8921)	18.1514 (0.8544)	0.064
2-week	6.9369 (0.4471)	6.4800 (0.4931)	0.300	24.6001 (1.3202)	22.3889 (1.0974)	0.090	21.4987 (0.8767)	20.1854 (1.6625)	0.293	20.3710 (0.8715)	18.9723 (0.9416)	0.132
4-week	8.0969 (0.6748)	7.6715 (0.6647)	0.480	28.9090 (1.0714)	27.8698 (1.3655)	0.358	24.7241 (2.2522)	23.8229 (2.8162)	0.687	22.3084 (1.3385)	21.4826 (1.1003)	0.456

Mean values, standard deviation (SD), and *p* values of each distance index are shown for each group (*C* control group; *CP* cleft palate group) at each time point in development.

*p < 0.05. Mandibular length: L3 (B–P); Mandibular height: L5 (B–C), L6 (B–G).

## Discussion

The treatment of cleft palate is dependent on surgical operations, but due to variations in surgical timing and methods, the development of maxillary structures and speech may be inadequate in some cleft palate patients. Over the past four decades, a multitude of investigators has sought to develop congenital and iatrogenic models of cleft palate in an attempt to develop new treatment strategies. The two key aspects are fetal intervention and clinical applications of gene therapy for the prevention of surgical complications, such as midfacial growth impairment, and VPI caused by muscle injury. Weinzweig et al. subsequently investigated the ultrastructural and functional aspects of the palate following in utero cleft repair to determine benefits that might be derived from fetal intervention^19,20^. In regards to in utero cleft palate repair, the authors focused on the reconstitution of the velar muscular sling, which is disrupted during the clefting process and remains abnormally inserted into the posterior edge of the palatal bone and along the bony cleft. Although repairing velar muscle did demonstrate some evidence of ultrastructural change compared with the control, these findings were significantly less pronounced than those observed in the unrepaired cleft muscle^21^. In contrast, midfacial hypoplasia and growth disturbances were demonstrated to have a significant influence of in utero palatoplasty in the cleft goats model repaired using a modified von Langenbeck technique^22^. Subsequent studies have indicated that the outcome of cleft palate repair is likely to be improved with clinical applications of gene therapy for the prevention of surgical complications. As experimental models, mice have made great contributions to the study of the mechanism of cleft palate, however, there has been no reliable animal model for the postnatal treatment study of cleft palate. Additionally, larger congenital animal models, such as beagles and sheep are more expensive and difficult to manipulate for cleft surgery. Thus, this study provides a method that may define and modify the mechanisms involved in gene therapy for the development of new treatment strategies in cleft palate.

The mechanism of the dexamethasone-induced cleft palate has not yet been clarified in literature, but the incidence of cleft palate caused by glucocorticoid is closely related to the genetic background of the animals^23^. In this experiment, the genetic background, physiological conditions, and living environment of the New Zealand pregnant rabbits were kept constant and were similar to that of Walker’s study. Therefore, the incidence of cleft at 60.4% was close to the result (61.5%) obtained by Walker using a similar dose of dexamethasone (Table 1)^13^. Additionally, the phenotype characteristics of newborn infants were also consistent with the results of the previous study^23^. We improved and adopted a stable feeding method to raise infant rabbits, although feeding through a gastric tube may be considered of some risks for infant rabbits, we found that this feeding method was reliable and can help the infants in the cleft group pass the lactation period in an active and healthy state. Besides, there was no difference in the weight, appearance, and behavior between the two groups (Fig. 2), which can prevent external bias factors in future research using this model.

After the observation of the cleft morphology, we determined that the cleft palate type was stable in our cleft model. Besides, the cleft model observed by surgical anatomy still presents some of the same characteristics to that of rodents with long soft palate and abundant muscle tissue, and similar studies have also shown that the soft palate muscles of rodents are more comparable to those of humans^9,10^. Meanwhile, the histological analysis reconfirmed the anatomical results and presented the characteristics of soft palate epithelium thickening, loose connective tissue hyperplasia, glandular degeneration, muscle tissue mass and arrangement disorder. Since palatal muscles play a key role in velopharyngeal function, and there is still a lack of studies involving postnatal soft palate muscle development in cleft palate patients, this model can be considered as the best model for the research about injury control and regeneration in the palatal muscle. The abnormal dental arch and disordered occlusion were seen in the cleft group is also a common occurrence in children with cleft palate, and were further confirmed in Micro-CT coronal sections, especially post-operative scans^24^. However, the mechanism of this deformity is still not clear, although injury during surgery has been deemed a key factor. Thus, a new treatment strategy focusing on the management of the abnormal dental arch is essential. Our cleft model could be beneficial in determining the origin of this issue by investigating the mechanism of injury in cleft palate surgery. Swolin-Eide found that prenatal dexamethasone exposure affects skeletal growth in rats but does not have an effect on bone mineralization. Other studies have shown that dexamethasone suppresses the Wnt/BMP pathways in osteoblasts^25,26^. In this study, the abnormal morphology and low bone density of nasal-maxillary structure were also obvious (Fig. 6). This is an area that may require further studies. Unlike a previous study that used spaniels as a congenital cleft palate model^27^, this study showed that there was no significant difference in the height, length, and width of the maxilla between the two groups at weeks 1, 2, and 4 (Tables 3, 4), and the difference in distance between bilateral pterygoid hamulus points could be considered as a normal difference that occurs in primary cleft palate. Furthermore, based on the assessment of mandibular development, the results showed that most mean values were generally lower in the cleft palate group, although only the length index at 1 week old was a statistically significant difference. Our results indicated that dexamethasone may affect cranial and maxillofacial development in multiple ways, which need to be confirmed by further research. So, compared with the existing animal models, the New Zealand rabbit model with congenital cleft palate can simulate human cleft palate to the greatest extent, thus providing continuous and reliable postnatal data for research of the developmental, morphological, and molecular levels.

In China, competency in cleft palate repair is required for oral and maxillofacial surgery training. At present, the training of cleft palate surgery is usually carried out through long-term theoretical learning, operation observation, and practice on simulated artificial models. It is a challenging procedure to teach and assess in the operating room because of the handling of delicate tissue, limited visibility in the infant oral cavity, and potential complications that can arise from subtle technical errors. Supervising surgeons are reluctant to allow trainees to perform this procedure, making it impractical for trainees to learn or be evaluated on live patients. As a result, training in cleft palate surgery takes time, requiring gradual increases in hands-on experience. In the USA and Canada, they have to develop a cleft palate simulator in cleft palate surgery to provide a platform for operative experience^28^. However, due to the limitation of artificial model materials, such training often fails to replicate real surgical situations. We believe that the animal experimental model introduced in this study could be used for the preclinical training of cleft palate surgery, especially for the dissection process. Although the premaxilla is quite long, the tissue structures and levels, the location of important anatomical sites, and anatomical defects are all like those of humans, making it suitable for training. Furthermore, the feeding method has proven to be reliable, as well as the stability of anatomical defects and maxillary development. Based on a large number of randomized controlled experiments, 12 months of age has been determined to be the ideal time for cleft palate repair in humans^29^. Accordingly, if a New Zealand rabbit model is used for training, we recommend 2-weeks-old rabbits be used as the sexual maturity of rabbits at 1 week was deemed equivalent to that of 7 months in humans.

To our knowledge, this study is the first to successfully establish and describe the postnatal dexamethasone-induced congenital cleft palate New Zealand rabbit model. This model has shown efficient cleft induction, reliable feeding method, and stable anatomical and developmental defects. Therefore, we believe that there are more research possibilities using this model and that it may be useful for the development of future cleft palate treatments, as well as for pre-clinical training.
